# MiR-130a-3p Inhibits PRL Expression and Is Associated With Heat Stress-Induced PRL Reduction

**DOI:** 10.3389/fendo.2020.00092

**Published:** 2020-03-03

**Authors:** Haojie Zhang, Ting Chen, Jiali Xiong, Baoyu Hu, Junyi Luo, Qianyun Xi, Qingyang Jiang, Jiajie Sun, Yongliang Zhang

**Affiliations:** ^1^Guangdong Province Key Laboratory of Animal Nutritional Regulation, National Engineering Research Center for Breeding Swine Industry, College of Animal Science, South China Agricultural University, Guangzhou, China; ^2^Guangdong Engineering & Research Center for Woody Fodder Plants, South China Agricultural University, Guangzhou, China

**Keywords:** miR-130a-3p, estrogen receptor α, heat stress, prolactin, GH3 cells

## Abstract

MicroRNAs (MiRNAs) play critical roles in the regulation of pituitary function. MiR-130a-3p has previously been found to be down-regulated in prolactinoma, but its roles in prolactin (PRL) regulation and the underlying mechanisms are still unclear. Heat stress has been shown to induce alteration of endocrine hormones and miRNAs expressions. However, there is limited information regarding the emerging roles of miRNAs in heat stress response. In this study, we transfected miR-130a-3p mimic into the pituitary adenoma cells (GH3 cells) to investigate the function of miR-130a-3p in regulating PRL. Our results showed that miR-130a-3p overexpression significantly decreased the PRL expression at both mRNA and protein levels. Subsequently, estrogen receptor α (ERα) was identified as a direct target of miR-130a-3p by bioinformatics prediction, luciferase reporter assay and western blotting assay. Furthermore, the inhibition of ERα caused by estrogen receptor antagonist significantly reduced the PRL expression. Overexpression of ERα rescued the suppressed expression of PRL caused by miR-130a-3p mimic. Besides, we also studied the effect of heat stress on PRL and miRNAs expressions. Interestingly, we found that heat stress reduced PRL and ERα expressions while it increased miR-130a-3p expression both *in vitro* and *in vivo*. Taken together, our results indicate that miR-130a-3p represses ERα by targeting its 3'UTR leading to a decrease in PRL expression, and miR-130a-3p is correlative with heat stress-induced PRL reduction, which provides a novel mechanism that miRNAs are involved in PRL regulation.

## Introduction

Prolactin (PRL) is a polypeptide hormone mainly synthesized and secreted from specialized cells of the anterior pituitary gland. PRL is best known for its effects on the development of mammary gland, synthesis of milk and maintenance of milk secretion (1, 2). However, PRL has over 300 separate biological activities (3). In reproduction, PRL performs multiple roles other than lactation, such as luteal function, testicular function, and maternal behaviors.

It is well-known that PRL-inhibiting factors (PIFs) and PRL-releasing factors (PRFs) supplied by hypothalamus control the production of PRL from the pituitary (4). Besides, estrogen is also a powerful hormone that regulates PRL synthesis and secretion (5–7). Estrogen exerts its function by binding to their designated receptors that can recognize the estrogen receptor element (ERE) (8) on the distal enhancer region of the PRL promoter. ERα was the first discovered estrogen receptor and has been the most thoroughly investigated (9). Furthermore, ERα has been reported as a nuclear receptor that interacts with Pit1 through an AF-2 domain to regulate PRL transcription (10).

MicroRNA (miRNAs) are small non-coding RNAs (20–25 bp) that negatively regulate gene expression at the posttranscription level (11). Mature miRNAs bind to the 3′-untranslated regions (3′-UTR) of target genes, resulting in translational repression or mRNA degradation (12). Increasing reports have indicated that miRNAs play important roles in pituitary hormone regulation. For example, miR-26b upregulates growth hormone (GH) expression by targeting lymphoid enhancer factor 1(LEF1) (13). MiR-375 is reported to regulate pro-opiomelanocortin (POMC) expression by targeting mitogen-activated protein kinase-8 (MAP3K8). MiR-200b/miR-429 are found to increase LH secretion by targeting ZEB (14), while miR-325-3p inhibits LH synthesis by targeting LHβ (15). MiR-132/212 and miR-361-3p directly or indirectly regulate gonadotropin-releasing hormone (GnRH)-induced FSH expression by targeting sirtuin1 (SIRT1) and FSHβ, respectively (16, 17). Besides, miR-9 has been found to promote PRL production by targeting dopamine D2 receptor (D2R) (18), which also suggests miRNAs can play a role in PRL regulation.

MiR-130a has been reported to be down-regulated in prolactinoma (19), indicating miR-130a may participate in the regulation of PRL. Although miR-130a has been reported to regulate cell proliferation and adipose differentiation (20–22), its role in the regulation of PRL remains unclear. Besides, in our work-in-progress research focused on the genome wide analysis of non-coding RNA in pituitaries of normal and heat-stressed sows, heat stress has already been found to affect miR-130a-3p expression as well as PRL expression. Moreover, studies have shown that miRNAs participate in heat stress response (23), and heat stress induces alteration of endocrine hormones (24). Taken together, data above may suggest a potential relationship among miR-130a-3p, PRL and heat stress.

In this study, we used GH3 cells as *in vitro* models to analyze the effects of miR-130a-3p on PRL expression and its regulatory mechanism in somatolactotrophs. We also investigated the influence of heat stress on PRL and miR-130a-3p expressions both *in vivo* and *in vitro*. The results indicate that miR-130a-3p represses ERα expression leading to an inhibition of PRL expression. Furthermore, heat stress can increase miR-130a-3p expression, hinting miR-130a-3p may be negatively correlated with the production of PRL under heat stress.

## Materials and Methods

### Animals and *in vivo* Experiments of Heat Stress

The female FVB mice (8 weeks old) were purchased from the Cyagen Biosciences (Suzhou, China) and housed at 25 ± 2°C, 12 h light (7:00 am−7:00 pm)-dark cycle with free access to water and food. After 1 week of adaptation, 15 mice weighing ~25 ± 2 g were divided randomly into three groups (*N* = 5) for repeated heat stress, including Day 0 (without exposure), Day 1(exposed one time), and Day 7(exposed seven times). Except the control group (Day 0), mice were placed in an artificial climate cabin with a temperature of 40°C and relative humidity of 50% for 2 h each time. During the light phrase, the heat exposures were conducted between 1:00 and 3:00 pm daily. At the end of heat exposures, the mice were euthanized immediately, and then the blood and pituitaries were collected. The samples of Day 0 group were collected at the same time as the Day 1 group. We centrifuged the blood at 3,000 × g for 20 min at 4°C to obtain serum for prolactin (PRL), adrenocorticotropic hormone (ACTH) and pro-opiomelanocortin (POMC) detection. Then, we extracted RNA and protein from the pituitaries to conduct quantitative real-time PCR (qPCR) and western blotting analysis. All animal experiments were conducted according to the laboratory animal management and welfare regulations approved by The Animal Ethics Committee of South China Agricultural University.

### Cell Culture

GH3 rat pituitary tumor cell line and HEK293T cells were purchased from American Type Culture Collection. GH3 cells were cultured in Dulbecco's modified Eagle's medium (DMEM)/F12 medium (Gibco, Grand Island, NY, USA) with 15% horse serum (Hyclone, Logan, UT, USA), 2.5% fetal bovine serum (FBS, Gibco, Carlsbad, CA, USA) and 1% penicillin and streptomycin (Gibco). HEK293T cells were cultured in DMEM (Gibco, Grand Island, NY, USA) supplemented with 10% FBS and 1% penicillin and streptomycin. The cells were both incubated at 37°C in a humidified atmosphere of 5% CO_2_. GH3 cells and HEK293T cells were used between passages 6–8.

### Prediction of Potential miRNA Gene Targets

The gene targets of miR-130a-3p were predicted using the web tools TargetScan (http://www.targetscan.org/vert_72/) and RNAhybrid (https://bibiserv.cebitec.uni-bielefeld.de/rnahybrid). On the web site of TargetScan, select a species first. Then, enter a microRNA name, such as miR-130a-3p. Finally, click the “submit” button, and the target genes of miR-130a-3p will be shown. In the online version of RNAhybrid, upload the miR-130a-3p and candidate targets sequences. Set the parameters and then click the “start calculation” button. Finally, the predicted results will be shown. In the prediction, only alignments with energies < −20 kcal/mol and no mismatch in the seed region were retained.

### MicroRNA Transfections

GH3 cells were seeded (3.0 × 10^5^ cells/ well) onto Poly-L-Lysine (PLL)-coated 12-well plates for 24 h before transfection and the medium was then replaced with fresh medium without serum and antibiotics. GH3 cells were transfected with 40 pmol/well miR-130a-3p mimic or negative control (NC) or miR-130a-3p inhibitor or inhibitor NC (Genepharma, Suzhou, China) with Lipofectamine 2000 (Invitrogen, Carlsbad, CA, USA) following the manufacturer's introduction. After 6 h of transfection, the medium was replaced and the cells were incubated in complete culture medium for 48 h before collection. The following oligos were used for cell transfection: rno-miR-130a-3p mimic (Forward: 5′-CAGUGCAAUGUUAAAAGGGCAU-3′, Reverse: 5′-GCCCUUUUAACAUUGCACUGUU-3′); NC (Forward: 3′-UUCUCCGAACGUGUCACGUTT-3′, Reverse: ACGUGACACGUUCGGAGAATT-3′); rno-miR-130a-3p inhibitor (5′-AUGCCCUUUUAACAUUGCACUG-3′); inhibitor NC (5′-CAGUACUUUUGUGUAGUACAA-3′).

### ICI Treatment and ERα Overexpression Vector Transfections

GH3 cells were seeded (3.0 × 10^5^ cells/ well) onto Poly-L-Lysine (PLL)-coated 12-well plates for 24 h with phenol red-free DMEM(Gibco, Grand Island, NY, USA) including 10% charcoal/dextran-treated FBS (Biological Industries, Kibbutz Beit Haemek, Israel), 1 % penicillin and streptomycin (Gibco/Invitrogen) for 24 h before treatment. The estrogen receptor antagonist ICI 182780 (ICI) (Abcam, Cambridge, MA, USA) for treatment was dissolved in dimethyl sulfoxide (DMSO, Sigma-Aldrich, St. Louis, MO, USA), diluted with the experimental medium, and added to the cell culture medium. The GH3 cells were treated with ICI (10 nM) or DMSO to obtain the vehicle control for 48 h.

The full coding sequence (CDS) of ERα was synthesized by Sangon Biotech (Shanghai, China) and cloned into pcDNA3.1 expression vector. The ERα overexpression vector was named pcDNA3.1- ERα. GH3 cells were seeded (3.0 × 10^5^ cells/ well) onto Poly-L-Lysine (PLL)-coated 12-well plates for 24 h before transfection and the medium was then replaced with fresh medium without serum and antibiotics. Then, the mixtures of NC and pcDNA3.1 or miR-130a-3p mimic and pcDNA3.1 or miR-130a-3p mimic and pcDNA3.1- ERα were transfected into GH3 cells with Lipofectamine 2000 (Invitrogen, Carlsbad, CA, USA). After 6 h of transfection, the medium was replaced and the cells were incubated in complete culture medium for 48 h before collection.

### Exposure of GH3 Cells to Heat Stress

GH3 cells were cultured in DMEM/F12 medium as previously described and seeded in 12-well plates at the density of 3.0 × 10^5^ cells per well. After 24 h culturing, the medium was replaced and one culture plate was transferred to 41°C (5% CO_2_, 95% air, 100% humidity) for another 24 h continuous culture. The control cells were still incubated at 37°C (5% CO_2_, 95% air, 100% humidity) for 24 h without any treatment.

### RNA Extraction and Quantitative Real-Time PCR (qRT-PCR) Analysis

The total RNA from pituitary or GH3 cells was isolated by TRIzol reagent (Invitrogen) according to the manufacturer's instructions. Then total RNA was treated with DNase I (Promega, Madison, WI, USA) to remove DNA contamination. The concentration and quality of RNA were determined by NanoDrop 2000 (Wilmington, DE, USA). 1 μg of total RNA was reverse transcribed using M-MLV reverse transcriptase (Promega, Madison, WI, USA). The specific stem-loop primer of miR-130a-3p and oligo(dT)_18_ primers were, respectively used for the reverse transcription of miR-130a-3p and mRNAs. The random primers were used for the reverse transcription of the small nuclear RNA U6. Quantitative real-time PCR (qRT-PCR) was performed on a Bio-Rad CFX96 Real-Time Detection System (Bio-Rad Laboratories, Inc., Hercules, CA) with the GoTaq qPCR Master Mix (Promega, Madison, WI, USA). The conditions of the qRT-PCR were as follows: 95°C for 5 min, followed by 40 cycles of 95°C for 15 s, 58°C for 30 s and 72°C for 30 s. The relative expressions of miR-130a-3p and genes were normalized respectively against that of the small nuclear RNA U6 and glyceraldehyde phosphate dehydrogenase (GAPDH). The relative expression was measured using the 2^−ΔΔCt^ method (25). All primer sequences were shown in Table S1.

### Western Blotting Analysis

GH3 cells or pituitaries were lysed in RIPA lysis buffer (Beyotime Institute of Biotechnology, Shanghai, China) containing 1 mM phenylmethanesulfonyl fluoride (PMSF). The concentration of protein was measured using the BCA Protein Assay Kit (Thermo Fisher Scientific, Waltham, MA) according to the manufacturer's instruction. Equivalent amounts of protein (20 μg) were separated by 10% SDS-PAGE gels and then transferred to polyvinylidene fluoride (PVDF) membranes (Millipore, Billerica, MA, USA). The membranes were blocked with 5% (w/v) non-fat dry milk in Tris-buffered saline containing 0.1% Tween 20 (TBST) for 2 h at room temperature and incubated in diluted primary antibodies overnight at 4°C. The primary antibodies used were rabbit polyclonal antibody against ESR1 (ERα) (1:1000; D222310; Sangon Biotech, Shanghai, China), goat polyclonal antibody against PRL (0.25 μg/ml; AF1445-SP; R&D system, Minneapolis, USA), rabbit polyclonal antibody against GAPDH (1:5000; bs-0755R; Bioss, Beijing, China) and rabbit polyclonal antibody against β-actin (1:5000; bs-0061R; Bioss, Beijing, China). The membranes were then washed six times (each for 5 min) in TBST, incubated with the HRP-conjugated secondary antibodies goat anti rabbit IgG and rabbit anti goat IgG (1:50000; Bioworld technology, Nanjing, China) for 1 h at room temperature and washed again with TBST. Finally, the membranes were incubated with Immobilon^TM^ Western Chemiluminescent HPR Substrate (Millipore, Burlington, USA) and scanned with a FlourChemMFluorescent Imaging System (ProteinSimple, Santa Clara, CA, USA). The protein band density was determined by the software Image J and normalized with corresponding GAPDH or β-actin band intensity.

### Cell Immunofluorescence

The Poly-L-Lysine (PLL)-coated coverslips were placed in 6-well plates. Then, GH3 cells were seeded onto the plate and transfected with miR-130a-3p mimic or negative control (NC) with Lipofectamine 2000 (Invitrogen) within 24 h. After transfection and cultivation, we took out the coverslips and washed the coverslips three times with PBS to remove culture medium. Next, we immersed the coverslips (cells face up) into 4% paraformaldehyde for 10 min and washed the coverslips three times with PBS. Finally, put the coverslips on filter paper (cells face up) to remove the liquid and allow them to dry 8–10 h. Next, immunofluorescence staining was performed on cells fixed on coverslips (26). The coverslips were incubated in 0.4% Triton X-100 for 10 min, and blocked in 10% goat serum for 30 min at room temperature. After that, the coverslips were incubated in ESR1 (ERα) antibody (1:200; NHA6712; Novogene, Beijing, China) and PRL polyclonal antibody (1 μg/ml; AF1445-SP; R&D system, Minneapolis, USA) at 4°C overnight. The next day, the coverslips were washed three times in PBS and incubated in FITC conjugated secondary antibodies (Bioss, Beijing, China) for 1 h and subsequently incubated in DAPI for 10 min. Finally, the fluorescence was detected and quantified using Nikon Eclipse Ti-s microscopy (Nikon Instruments, Japan).

### Hormone Assay

Mice serum PRL, ACTH, and POMC contents were measured using PRL Elisa Kit, ACTH Elisa Kit, and POMC Elisa Kit according to the manufacturer's instructions, respectively. All the Elisa Kits were purchased from Shanghai Enzyme-linked Biotechnology Co., Ltd. (Shanghai, China). The minimum detectable concentration was 1.25 ng/mL for PRL, 2.5 pg/mL for ACTH and 0.25 ng/mL for POMC. The intra-assay and inter-assay coefficients of variation were <10%.

### Luciferase Reporter Assay

The mutant, deleted and wild-type seed region of ERα 3'UTR containing XhoI and XbaI sites were cloned by overlap PCR and the primers were listed in Table S2. The PCR products were inserted in the pmirGLO Vector (Promega, Madison, USA) to construct three dual-luciferase reporter plasmids. HEK293T is easily transfectable and unproblematic for luciferase expression. The cell line is often used for the luciferase-assay of miRNA and its target genes or circRNA and target miRNAs (27, 28). Therefore, we used HEK293T cells to do the luciferase-assay. The HEK293T cells (4 × 10^4^ per well) were plated in a 96-well plate and transfected with 3 pmol miR-130a-3p mimic/NC and 100 ng constructs using Lipofectamine 2000. Cells were collected after 48 h transfection, and the luciferase activity was measured by the Dual-GLO luciferase reporter assay system (Promega, Madison, USA) according to the manufacturer's introduction.

### Statistical Analysis

*In vitro* experiments, all values are expressed as mean ± standard error of the mean (S.E.M) based on data obtained from at least four samples per group in a single experiment. *In vivo* experiments, all values are presented as mean ± S.E.M or mean ± standard deviation (SD) based on data obtained from at least three animals per group. The statistical analysis was performed using SPSS software (Chicago, IL, USA). The student's *t*-test was used to compare the differences between the treatment and control samples, and one-way analysis of variance (ANOVA) followed by a Tukey's HSD test was used when more than two groups were compared. *P* < 0.05 was considered to be statistically significant.

## Results

### MiR-130a-3p Overexpression Reduces PRL Expression in GH3 Cells

**Figure 1:**
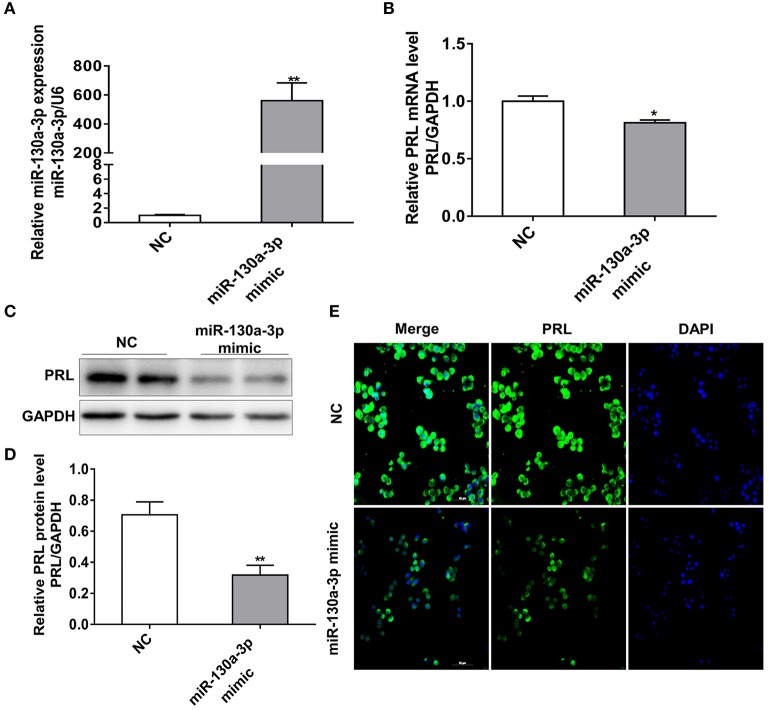
MiR-130a-3p overexpression reduces PRL expression in GH3 cells. GH3 cells were transfected with miR-130a-3p mimic and NC and then the expressions of miR-130a-3p and PRL were analyzed. **(A)** The expression level of miR-130a-3p was detected by quantitative real-time PCR (qRT-PCR). U6 snRNA was used to normalize the miRNA expression. Data are presented as mean ± S.E.M of *n* = 6 samples per group (*P < 0.05 by *t*-test). **(B)** The expression level of PRL mRNA was detected by qRT-PCR. GAPDH was used to normalize each gene expression. Data are presented as mean ± S.E.M of *n* = 6 samples per group (*P < 0.05 by *t*-test). **(C)** The protein level of PRL in GH3 cells was analyzed by western blotting. GAPDH was used as loading control. **(D)** Quantitation of the PRL protein level. Data are presented as mean ± S.E.M of *n* = 4 samples per group (*P < 0.05 by *t*-test). **(E)** The protein level of PRL was detected by ICC after GH3 cells transfected with miR-130a-3p mimic and NC. Scale bar, 50 μM. **P < 0.01.

To investigate whether miR-130a-3p plays a role in regulating PRL expression, we transfected GH3 cells with miR-130a-3p mimic or negative control (NC) and detected the mRNA and protein expressions of PRL. As expected, the miR-130a-3p mimic robustly increased miR-130a-3p levels compared to the negative control (NC) (Figure 1A). Meanwhile, treatment with miR-130a-3p mimic reduced the expression of PRL not only at mRNA level (Figure 1B) but also at protein level in GH3 cells (Figures 1C–E). Since GH3 cells can also produce GH, we tested the expression of GH in order to verify the specific role of miR-130a-3p in PRL. The results showed miR-130a-3p mimic had no significant effect on GH mRNA (Figure S1A) and protein (Figures S1B,C) expressions. These data demonstrate that miR-130a-3p inhibits PRL expression in GH3 cells.

### MiR-130a-3p Directly Targets ERα 3'UTR and Inhibits ERα Expression

**Figure 2:**
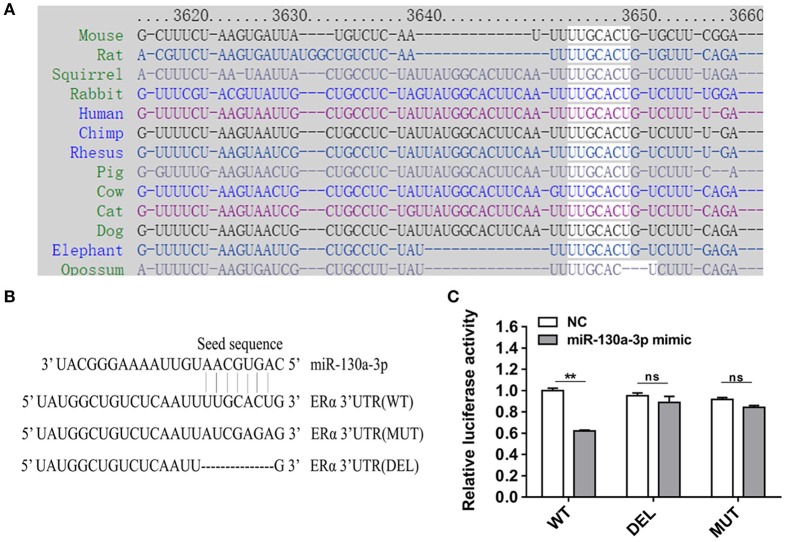
MiR-130a-3p directly targets ERα. **(A)** The predicted miR-130a-3p binding site in ERα 3'UTR is evolutionarily conserved. **(B)** Partial ERα 3'UTR sequences in wild-type (WT), mutant (MUT), and deleted (DEL) reporter vectors. The seed sequence binding sites of miR-130a-3p were replaced or deleted in MT and DEL. **(C)** Dual-luciferase assay following co-transfection of the ERα 3'UTR (WT/MUT/DEL) reporter vectors with miR-130a-3p mimic or negative control (NC) in HEK293T cells. Data are presented as mean ± S.E.M of *n* = 8 samples per group (**P < 0.01, ns, not significant by *t*-test).

**Figure 3:**
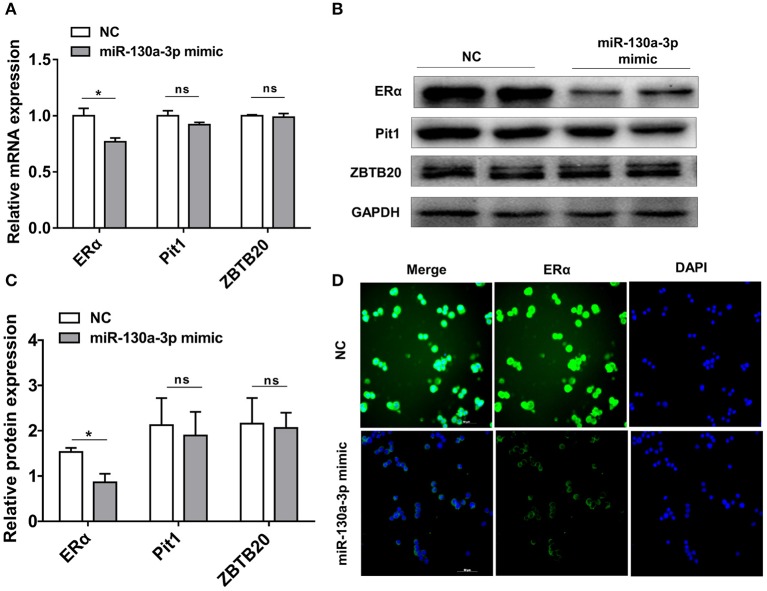
MiR-130a-3p overexpression reduces ERα expression in GH3 cells. GH3 cells were transfected with miR-130a-3p mimic and negative control (NC) and then the expressions of ERα and genes related to PRL regulation were analyzed. **(A)** The expression levels of ERα, Pit1, and ZBTB20 were detected by quantitative real-time PCR (qRT-PCR). GAPDH was used to normalize each gene expression. Data are presented as mean ± S.E.M of *n* = 6 samples per group (*P < 0.05 by *t*-test). **(B)** The protein levels of ERα, ZBTB20, and Pit1 were analyzed by western blotting. GAPDH was used as loading control. **(C)** Quantitation of ERα, ZBTB20, and Pit1 protein levels. Data are presented as mean ± S.E.M of *n* = 4 samples per group (*P < 0.05 by *t*-test). **(D)** The protein level of ERα was detected by ICC after GH3 cells transfected with miR-130a-3p mimic and NC. Scale bar, 50 μM, ns, not significant.

To determine the potential target through which miR-130a-3p exerts its effects on GH3 cells, we used TargetScan and RNAhybrid to predict and screen miR-130a-3p target genes related to PRL regulation. Interestingly, we found ERα was a putative target of miR-130a-3p. The predictive software showed that ERα 3'UTR contained putative binding sites for miR-130a-3p seed sequence. The targeting relationship was conserved among different species (Figure 2A). In order to verify whether miR-130a-3p directly binds to the 3'UTR of ERα, the 3'UTR sequence of ERα harboring the predicted binding sites for miR-130a-3p was inserted downstream of the luciferase reporter vector pmirGLO. ERα 3'UTR sequences with a mutation or deletion in the seed sequence were also designed and inserted (Figure 2B). These constructs were named as WT, MUT, and DEL, respectively. HEK 293T cells were co-transfected with the constructed vectors (WT, MUT, and DEL) as well as miR-130a-3p mimic or NC. The relative luciferase activity measurement revealed that miR-130a-3p mimic significantly reduced the luciferase activity of the WT reporter, but had no effect on the mutant and deleted ERα-3'UTR reporters (Figure 2C). These results suggest that ERα is a direct target of miR-130a-3p. Then, we analyzed the effect of miR-130a-3p mimic on the expressions of ERα mRNA and protein. The results showed miR-130a-3p mimic decreased ERα mRNA level in GH3 cells as assessed by qRT-PCR (Figure 3A). The protein level of ERα determined by both western blotting and cell immunofluorescence was also significantly reduced when miR-130a-3p mimic was transfected (Figures 3B–D). Moreover, we also examined the expressions of Zinc finger and BTB domain-containing protein 20 (ZBTB20) and pituitary specific transcription factor 1 (Pit1), which are two key transcription factors of PRL. MiR-130a-3p mimic did not significantly affect ZBTB20 and Pit1 mRNA and protein levels (Figures 3B,C). These findings indicate ERα is a direct target of miR-130a-3p.

### Inhibition of ERα Reduces the Expression of PRL

**Figure 4:**
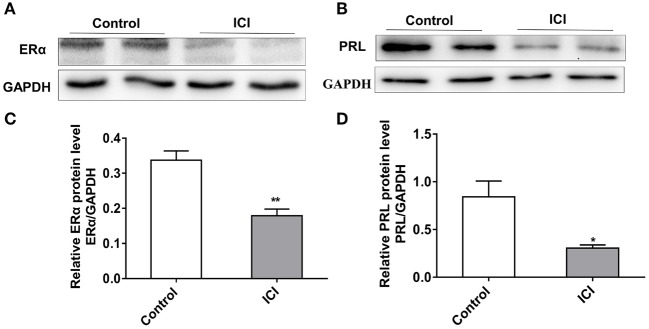
Inhibition of ERα reduces the expression of PRL. GH3 cells were treated with 10 nM ICI 182780 (ICI) or the vehicle control for 48 h. The protein expressions of ERα and PRL were analyzed. **(A)** The protein expression level of ERα was detected by western blotting. GAPDH was used as loading control. **(B)** The protein expression level of PRL was detected by western blotting. GAPDH was used as loading control. **(C)** Quantitation of ERα protein level. Data are presented as mean ± S.E.M of *n* = 4 samples per group (*P < 0.05 by *t*-test). **(D)** Quantitation of PRL protein level. Data are presented as mean ± S.E.M of *n* = 4 samples per group (*P < 0.05 by *t*-test). **P < 0.01.

To characterize the effect of ERα on PRL expression, we inhibited the expression of ERα in GH3 cells using the estrogen receptor antagonist ICI 182780 (ICI). The protein level of ERα significantly decreased after ICI treatment (Figures 4A,C), and knockdown of ERα significantly reduced the protein expression level of PRL (Figures 4B,D). These results suggest that inhibition of ERα reduces PRL expression.

### Overexpression of ERα Rescues miR-130a-3p-Inhibited Expression of PRL

**Figure 5:**
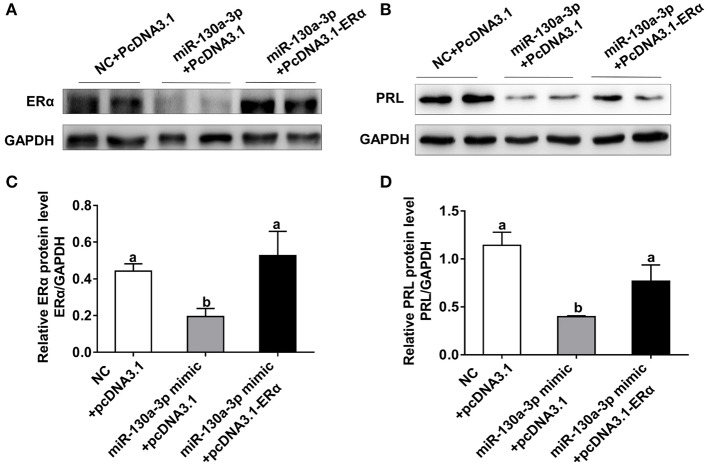
Overexpression of ERα rescues miR-130a-3p-inhibited expression of PRL. The GH3 cells were transfected with the miR-130a-3p mimic and the ERα overexpressing vector. The rescue efficiency of ERα in the GH3 cells was confirmed by western blotting. **(A)** The protein expression level of ERα was detected by western blotting. GAPDH was used as loading control. **(B)** The protein expression level of PRL was detected by western blotting. GAPDH was used as loading control. **(C)** Quantitation of ERα protein level. Data are presented as mean ± S.E.M of *n* = 4 samples per group (*P* < 0.05 by ANOVA). **(D)** Quantitation of PRL protein level. Data are presented as mean ± S.E.M of *n* = 4 samples per group (*P* < 0.05 by ANOVA).

To assess whether ERα is a key functional target of miR-130a-3p in GH3 cells, we performed rescue experiments with ERα overexpression vector (PcDNA3.1-ERα). The miR-130a-3p-inhibited protein expression of ERα was rescued by ERα overexpression vector (Figures 5A,C). Meanwhile, overexpression of ERα rescued the protein inhibition of PRL caused by miR-130a-3p (Figures 5B,D). Therefore, the above results indicate that ERα is functionally relevant to the miR-130a-3p mediated regulation of PRL expression.

### MiR-130a-3p Inhibitor Rescues MiR-130a-3p Mimic Induced Reduction of PRL and ERα

As the overexpression of miR-130a-3p resulted in a significant decrease in ERα and PRL expressions, we also tested whether miR-130a-3p inhibitor rescued this effect. The results showed that miR-130a-3p inhibitor transfection had no significant effect on ERα and PRL expressions (Figures S2A–C). However, when we co-transfected GH3 cells with 40 pmol mimic and 80 pmol inhibitor, the levels of miR-130-3p (Figure S2D), ERα (Figures S2E,G,H), and PRL (Figures S2F,G,I) were all rescued. These results may be explained by the fact that GH3 cells expressed relatively low level of endogenous miR-130a-3p, therefore the effect of miR-130a-3p inhibitor was limited under the normal condition but significantly efficient under the high expression of miR-130a-3p. Taken together, the results provided evidences that miR-130a-3p is involved in ERα and PRL regulation.

### Heat Stress Decreases PRL and ERα Expressions but Increases MiR-130-3p Expression in Mice Pituitary

**Figure 6:**
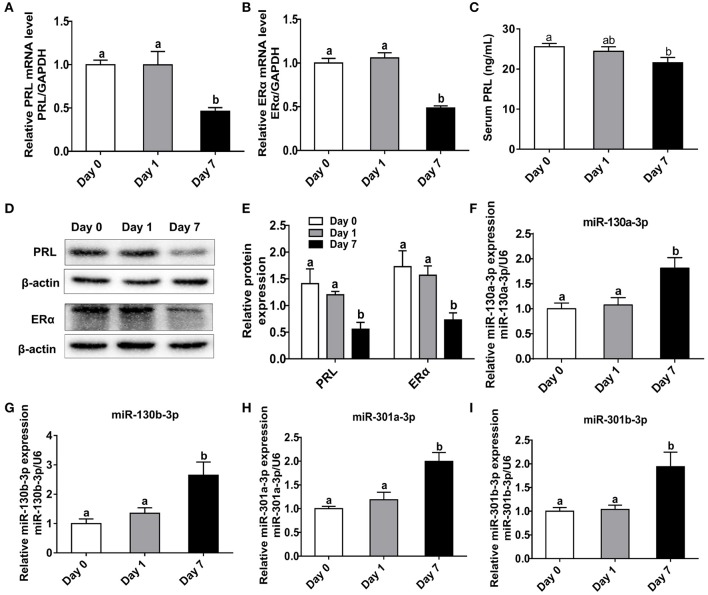
Heat stress reduces PRL and ERα expressions as well as increases miR-130a-3p expression in the pituitary gland. The mice of treated groups were placed in 40°C for 2 h each time, and the stimulus, respectively lasted 1 and 7 days. The mice in control group were fed as normal in 25°C. Then the expressions of PRL and ERα in the pituitary gland were analyzed. **(A–B)** The mRNA levels of PRL **(A)** and ERα **(B)** in the pituitary gland of three groups were analyzed by quantitative real-time PCR (qRT-PCR). GAPDH was used to normalize each gene expression. Data are presented as mean ± S.E.M of *n* = 5 animals per group. Bars that share different letter are significantly different (*P* < 0.05 by ANOVA). **(C)** The serum PRL concentration was detected by Elisa assay. Data are presented as mean ± S.E.M of *n* = 5 animals per group. Bars that share different letter are significantly different (*P* < 0.05 by ANOVA). **(D)** The protein levels of PRL and ERα in the pituitary gland were analyzed by western blotting. β-actin was used as loading control. **(E)** Quantitation of PRL and ERα protein levels. Data are presented as mean ± SD of *n* = 3 animals per group. Bars that share different letter are significantly different (*P* < 0.05 by ANOVA). The expression levels of miR-130a-3p **(F)**, miR-130b-3p **(G)**, miR-301a-3p **(H)**, and miR-301b-3p **(I)** were detected by qRT-PCR. U6 snRNA was used to normalize each miRNA expression. Data are presented as mean ± S.E.M of *n* = 5 animals per group. Bars that share different letter are significantly different (*P* < 0.05 by ANOVA).

In order to investigate the relationships among heat stress, PRL and ERα, we analyzed the expressions of PRL and ERα in mice exposed to heat stress. Results showed transient heat stress at Day 1 had no effect on pituitary PRL and ERα expressions, while the repeated heat stress significantly decreased pituitary PRL and ERα mRNA levels (Figures 6A,B) as well as protein levels (Figures 6D,E) at Day 7. In addition, we measured the contents of PRL, ACTH, and POMC in mice serum. The results showed the PRL level was significantly decreased at Day 7 (Figure 6C), while the ACTH level increased at Day 7 (Figure S3A), and the POMC level did not change (Figure S3B). Next, we tested miR-130a-3p expression of mice pituitary under heat stress. Interestingly, miR-130a-3p expression did not change at Day 1, but significantly increased at Day 7 (Figure 6F). We also examined the expressions of miR-130a-3p family members and achieved similar results (Figures 6G–I). These results indicate that long-term heat exposure reduces pituitary PRL and ERα expressions, and increases the expression of miR-130a-3p, as well as its family members.

### Heat Stress Increases MiR-130a-3p Expression and Reduces ERα and PRL Expressions in GH3 Cells

**Figure 7:**
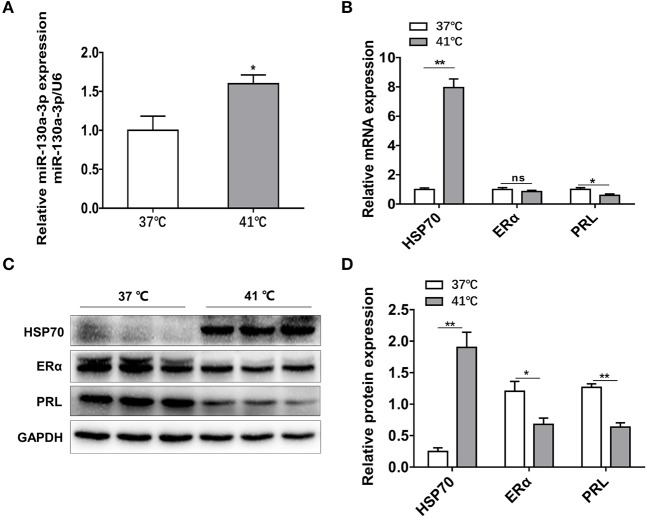
Heat stress increases miR-130a-3p expression, reduces PRL and ERα expressions in GH3 cells. GH3 cells were separately cultured in 37 or 41°C for 24 h, after that, miR-130a-3p, PRL, and ERα expressions were analyzed. **(A)** The expression level of miR-130a-3p was detected by quantitative real-time PCR (qRT-PCR). U6 snRNA was used to normalize the miRNA expression. Data are presented as mean ± S.E.M of *n* = 4 samples per group (*P < 0.05 by *t*-test). **(B)** The expression levels of HSP70, ERα, and PRL mRNAs in GH3 cells were detected by qRT-PCR. GAPDH was used to normalize each gene expression. Data are presented as mean ± S.E.M of *n* = 4 samples per group (*P < 0.05, ns, not significant by *t*-test). **(C)** The protein levels of HSP70, ERα, and PRL in GH3 cells were analyzed by western blotting. GAPDH was used as loading control. **(D)** Quantitation of HSP70, ERα, and PRL protein levels. Data are presented as mean ± S.E.M of *n* = 4 samples per group (*P < 0.05 by *t*-test). **P < 0.01.

To further confirm the results above *in vivo*, GH3 cells were separately placed at 37 and 41°C for 24 h and the related genes expressions were measured. The mRNA and protein levels of HSP70 were significantly increased under heat exposure (Figures 7B–D), indicating that the cellular model of heat stress was successfully established. Then, miR-130a-3p expression was found to be significantly upregulated in GH3 cells at 41°C compared to control (Figure 7A). Furthermore, we also found that expressions of ERα and PRL were inhibited at 41°C (Figures 7B–D). The results of *in vitro* tests were consistent with *in vivo* experiments, which suggests a correlative relationship among miR-130a-3p, PRL, and heat stress.

## Discussion

The functions of miRNAs on pituitary hormone regulation has been increasingly reported. Since miR-130a-3p was down-regulated in prolactinoma (19), a pituitary adenoma that produces an excessive amount of PRL, we speculate that miR-130a-3p may be involved in PRL regulation. In the present study, we transfected GH3 cells with miR-130a-3p mimic and found miR-130a-3p overexpression significantly decreased PRL expression as well as ERα expression. The bioinformatics analysis predicted that miR-130a-3p might target ERα. The luciferase reporter assay confirmed this prediction. Moreover, in GH3 cells, inhibition of ERα induced the reduction of PRL expression, and overexpression of ERα rescued miR-130a-3p's inhibition on ERα and PRL expressions. These above results suggest that miR-130a-3p represses ERα by targeting its 3'UTR leading to a decrease in PRL expression. In this study, we also revealed that the expression of pituitary miR-130a-3p was increased under heat stress both *in vivo* and *in vitro*. Meanwhile, PRL and ERα were significantly decreased. Therefore, the results indicate that miR-130a-3p may be a heat stress-related miRNA and plays a role in heat stress response.

In previous studies, miR-130a-3p was usually reported to act as a tumor suppressor in various type of cancers (21, 29, 30) or a repressor in adipocyte tissue inhibiting the induction of preadipocytes into mature adipocytes (31). In this study, we firstly confirmed miR-130a-3p inhibited PRL expression, suggesting that miR-130a-3p may be involved in pituitary hormone regulation. Previous report has shown that miR-9 regulates prolactin production through D2 receptor (18). Our study showed that miR-130a-3p targeted ERα and led to a decrease of PRL expression. It has been reported that ligand-bound ERs (ERα or ERβ) can form dimers which directly act on the estrogen response elements (EREs) in the promoter regions of estrogen-regulated genes (e.g., PRL) and activate the genes transcription (32). In our study, we found the degradation of ERα induced PRL expression inhibition in GH3 cells, which is consistent with previous reports (33). Besides, overexpression of ERα rescued the inhibition of miR-130a-3p on ERα and PRL expressions. Therefore, our results indicate that ERα is functionally relevant for the miR-130a-3p mediated regulation of PRL expression. There are also some other transcription factors regulating PRL expression. For instance, Pit1, a pituitary-specific transcription factor, regulates the transcription of genes encoding hormone products, such as PRL and GH (34). ZBTB20, a transcription factor of PRL, increases PRL expression and secretion in GH3 cells (35). In the present study, both mRNA and protein levels of Pit1 and ZBTB20 were not changed by miR-130a-3p mimic, which indicates that Pit1 and ZBTB20 may exert no functions on the inhibition of miR-130a-3p on PRL expression.

Although the miR-130a-3p inhibitor rescued the reduction of ERα and PRL induced by miR-130a-3p mimic, the inhibitor alone had no significant changes relative to the control. This similar situation of miRNAs inhibitor also happened in previous studies (36, 37). One possible reason may be that the endogenous miR-130a-3p level was low in GH3 cells, which limited the effect of miR-130a-3p inhibitor (38). Another alternative reason could be that the inhibitor has been reported to sequester miRNAs without resulting in degradation (39), which may induce an off-target effect.

Heat stress is well-known to affect endocrine gland and the release of hormones (24), such as cortisol, thyroxine, reproductive hormones, growth hormone as well as PRL. However, its influence on PRL across different species is still controversial. Some studies have reported heat stress increased serum/plasma PRL concentration in human (40, 41) and ruminants (42, 43). Other studies have shown that heat stress did not affect PRL concentration in pigs (44, 45). In addition, studies also indicated that heat stress decreased PRL concentration in pigs (46) and mice (47). In our *in vivo* study, we found that heat stress decreased the PRL concentration of the mouse serum, which is consistent with a previous report related to heat-stressed mice (47). In our *in vitro* study, we found heat stress decreased PRL expression in rat GH3 cells. Since the animal models and cell lines used in our experiments are both rodents, the observed PRL decrease under heat stress may be species-specific. Besides, the inconsistency of PRL alteration among different species under heat stress may result from different physiological states, different sampling frequencies, or different degrees of heat stress (acute and chronic). The lower expression of PRL under heat stress may result in a decrease in lactation performance (47). It has been reported that PRL is related to testosterone secretion in normal adult men (48). Therefore, the reduced PRL expression under heat stress may also be a reason of the reductions in the fertility of organisms during summer. One main feature of the heat stress is the activation of the hypothalamus-pituitary-gonadal (HPA) axis leading to the increase of ACTH (49). We also detected the increased serum ACTH under heat stress (Figure S3A), which indicates that the HPA axis of mice is activated in the process of heat exposure. POMC is well-known to be increased under stress reaction. However, we did not detect any changes in serum POMC under heat stress. This may because that there is no change in the gene expression of POMC in the pituitary, which also occurred in heat stress experiments in rats (50). Additionally, it is indicated that heat stress regulates CRH expression and HPA axis activity by affecting miR-212 (23). In our study, we found that miR-130a-3p inhibited PRL expression. Heat stress increased miR-130a-3p expression and inhibited PRL as well as ERα expression both *in vivo* and *in vitro*. Resultantly, we believe that miR-130a-3p may be a heat stress-related miRNA and plays a role in heat stress response.

Intriguingly, the expression of miR-130a-3p, as well as the expressions of other miR-130a family members including miR-130b-3p, miR-301a-3p, and miR-301b-3p, were significantly up-regulated by heat stress. Since our findings show that miR-130a-3p represses ERα expression and leads to a decrease of PRL expression, it is deducible that the increased level of miR-130a-3p under heat stress may play a role in heat stress-mediated PRL and ERα inhibition. It has been reported that some miRNAs can be grouped as a family based on the sequence conservation at the 5' end of miRNAs (51, 52). These miRNA family members could repress a common set of targets and exert equivalent function (53). For the reason that miR-130b-3p, miR-301a-3p, and miR-301b-3p share complete sequence identity with miR-130a-3p in the seed region, it is possible that they may repress a common target and then function together with miR-130a-3p in heat stress-mediated PRL and ERα inhibition.

In summary, our present study indicates that miR-130a-3p represses ERα by targeting its 3'UTR leading to a decrease in PRL expression, and the increased miR-130a-3p is possibly involved in heat stress-induced PRL reduction. These findings provide a novel mechanism that miRNAs are involved in PRL regulation, as well as play a possible role in heat stress-induced PRL reduction.

## Data Availability Statement

All datasets generated for this study are included in the article/Supplementary Material.

## Ethics Statement

The animal study was reviewed and approved by The Animal Ethics Committee of South China Agricultural University.

## Author Contributions

YZ and JS were responsible for the main experimental concept and design. The experiments were performed by HZ, TC, JX, and BH. HZ, JL, QX, and QJ performed the data analyses and contributed reagents. The manuscript was written by HZ, JS, and YZ. All the authors approved the final version.

### Conflict of Interest

The authors declare that the research was conducted in the absence of any commercial or financial relationships that could be construed as a potential conflict of interest.

## Supplementary Material

Figure S1 The effect of miR-130a-3p on GH expression. GH3 cells were transfected with miR-130a-3p mimic and NC and then the expression of GH was analyzed. **(A)** The mRNA expression level of GH was detected by quantitative real-time PCR (qRT-PCR). GAPDH was used to normalize each gene expression. Data are presented as mean ± S.E.M of *n* = 6 samples per group (^*^*P* < 0.05 by *t*-test). **(B)** The protein level of GH in GH3 cells was analyzed by western blotting. GAPDH was used as loading control. **(C)** Quantitation of the GH protein level. Data are presented as mean ± S.E.M of *n* = 4 samples per group (^*^*P* < 0.05 by *t*-test).

Figure S2 MiR-130a-3p inhibitor rescues miR-130a-3p mimic induced reduction of PRL and ERα. **(A)** GH3 cells were transfected with miR-130a-3p inhibitor or the inhibitor negative control (inhibitor NC), and the relative expressions of miR-130a-3p, PRL, and ERα were detected by quantitative real-time PCR (qRT-PCR). U6 snRNA and GAPDH were, respectively used to normalize the miRNA expression or gene expression. Data are presented as mean ± S.E.M of *n* = 4 samples per group (^*^*P* < 0.05, ns, not significant by *t*-test). **(B)** GH3 cells were transfected with miR-130a-3p inhibitor or the inhibitor NC, and the protein levels of ERα and PRL were analyzed by western blotting. GAPDH was used as loading control. **(C)** Quantitation of ERα and PRL protein levels. Data are presented as mean ± S.E.M of *n* = 4 samples per group (^*^*P* < 0.05, ns, not significant by *t*-test). **(D)** GH3 cells were transfected with NC, miR-130a-3p mimic or the mixture of miR-130a-3p mimic and inhibitor. The expression of miR-130a-3p was detected by qRT-PCR. U6 snRNA was used to normalize the miRNA expression. Data are presented as mean ± S.E.M of *n* = 4 samples per group. Bars that do not share the same letter are significantly different (*P* < 0.05 by ANOVA). **(E,F)** The expression levels of ERα **(E)** and PRL **(F)** mRNA were detected by qRT-PCR. GAPDH was used to normalize each gene expression. Data are presented as mean ± S.E.M of *n* = 4 samples per group. Bars that do not share the same letter are significantly different (*P* < 0.05 by ANOVA). **(G)** The protein levels of ERα and PRL in GH3 cells were analyzed by western blotting. GAPDH was used as loading control. **(H,I)** Quantitation of ERα **(H)** and PRL **(I)** protein levels. Data are presented as mean ± S.E.M of *n* = 4 samples per group. Bars that do not share the same letter are significantly different (*P* < 0.05 by ANOVA).

Figure S3 The serum concentrations of ACTH and POMC. The mice of treated groups were placed in 40°C for 2 h each time, and the stimulus, respectively lasted 1 and 7 days. The mice in control group were fed as normal in 25°C. **(A)** The serum ACTH concentration was detected by Elisa assay. Data are presented as mean ± S.E.M of *n* = 5 animals per group. Bars that share different letter are significantly different (*P* < 0.05 by ANOVA). **(B)** The serum POMC concentration was detected by Elisa assay. Data are presented as mean ± S.E.M of *n* = 5 animals per group. Bars that share different letter are significantly different (*P* < 0.05 by ANOVA).
