# Supplementary figures and images for: MiR-130a-3p Inhibits PRL Expression and Is Associated With Heat Stress-Induced PRL Reduction
Source: Front Endocrinol (Lausanne). 2020 Mar 3;11:92. doi: 10.3389/fendo.2020.00092 (PMC7062671; doi:10.3389/fendo.2020.00092)

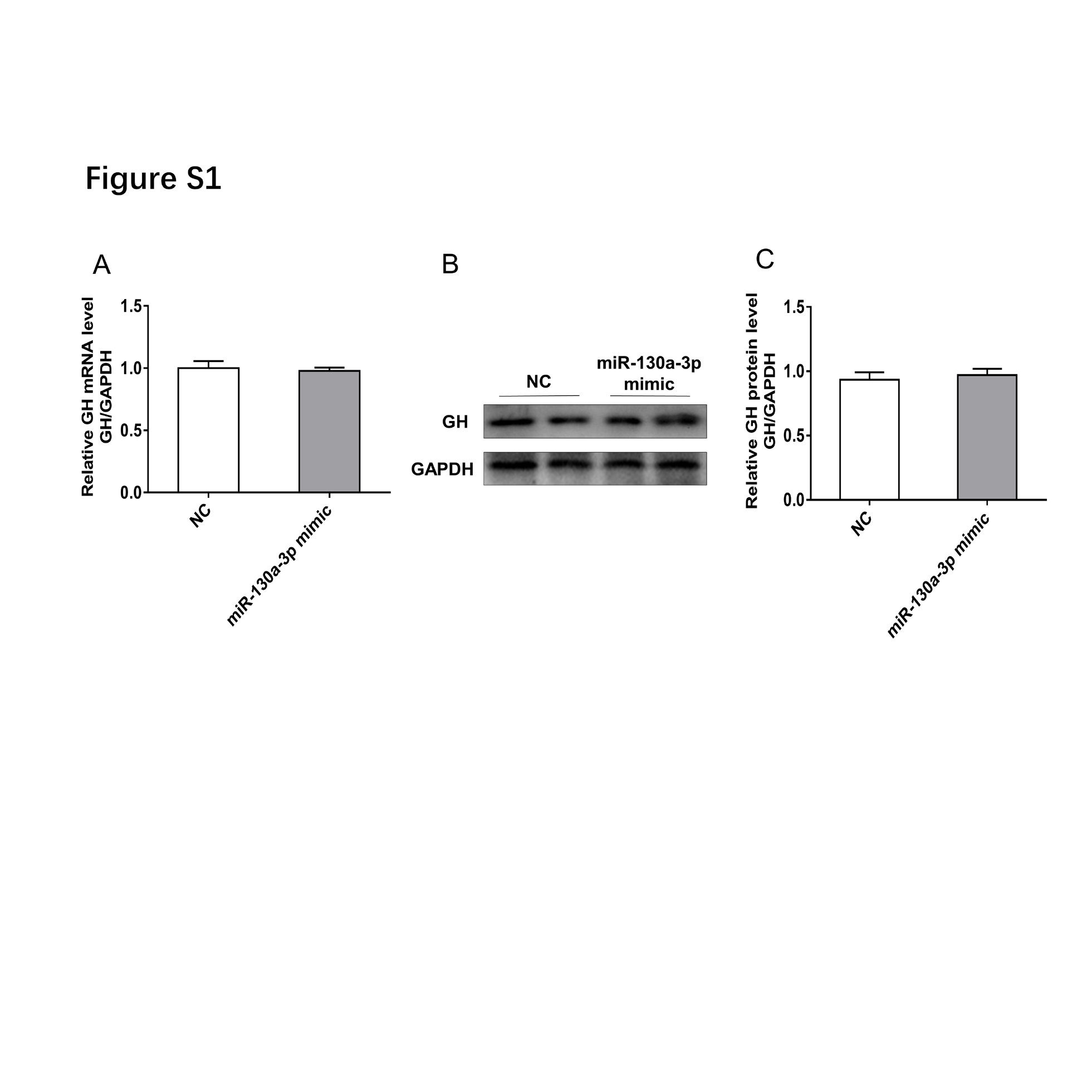

Supplement: Figure S1 — The effect of miR-130a-3p on GH expression. GH3 cells were transfected with miR-130a-3p mimic and NC and then the expression of GH was analyzed. (A) The mRNA expression level of GH was detected by quantitative real-time PCR (qRT-PCR). GAPDH was used to normalize each gene expression. Data are presented as mean ± S.E.M of n = 6 samples per group (*P < 0.05 by t-test). (B) The protein level of GH in GH3 cells was analyzed by western blotting. GAPDH was used as loading control. (C) Quantitation of the GH protein level. Data are presented as mean ± S.E.M of n = 4 samples per group (*P < 0.05 by t-test). [file Image_1.tif]

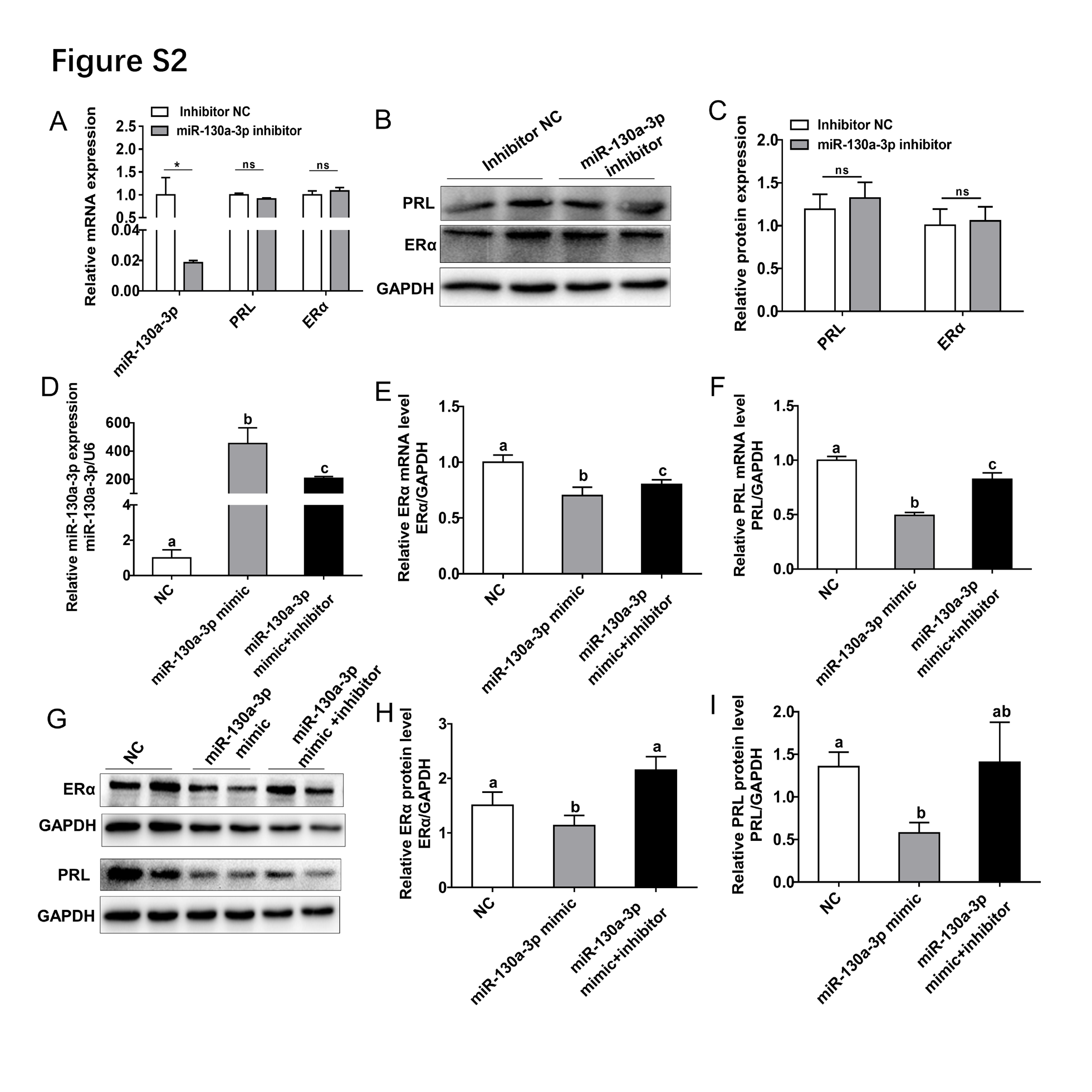

Supplement: Figure S2 — MiR-130a-3p inhibitor rescues miR-130a-3p mimic induced reduction of PRL and ERα. (A) GH3 cells were transfected with miR-130a-3p inhibitor or the inhibitor negative control (inhibitor NC), and the relative expressions of miR-130a-3p, PRL, and ERα were detected by quantitative real-time PCR (qRT-PCR). U6 snRNA and GAPDH were, respectively used to normalize the miRNA expression or gene expression. Data are presented as mean ± S.E.M of n = 4 samples per group (*P < 0.05, ns, not significant by t-test). (B) GH3 cells were transfected with miR-130a-3p inhibitor or the inhibitor NC, and the protein levels of ERα and PRL were analyzed by western blotting. GAPDH was used as loading control. (C) Quantitation of ERα and PRL protein levels. Data are presented as mean ± S.E.M of n = 4 samples per group (*P < 0.05, ns, not significant by t-test). (D) GH3 cells were transfected with NC, miR-130a-3p mimic or the mixture of miR-130a-3p mimic and inhibitor. The expression of miR-130a-3p was detected by qRT-PCR. U6 snRNA was used to normalize the miRNA expression. Data are presented as mean ± S.E.M of n = 4 samples per group. Bars that do not share the same letter are significantly different (P < 0.05 by ANOVA). (E,F) The expression levels of ERα (E) and PRL (F) mRNA were detected by qRT-PCR. GAPDH was used to normalize each gene expression. Data are presented as mean ± S.E.M of n = 4 samples per group. Bars that do not share the same letter are significantly different (P < 0.05 by ANOVA). (G) The protein levels of ERα and PRL in GH3 cells were analyzed by western blotting. GAPDH was used as loading control. (H,I) Quantitation of ERα (H) and PRL (I) protein levels. Data are presented as mean ± S.E.M of n = 4 samples per group. Bars that do not share the same letter are significantly different (P < 0.05 by ANOVA). [file Image_2.tif]

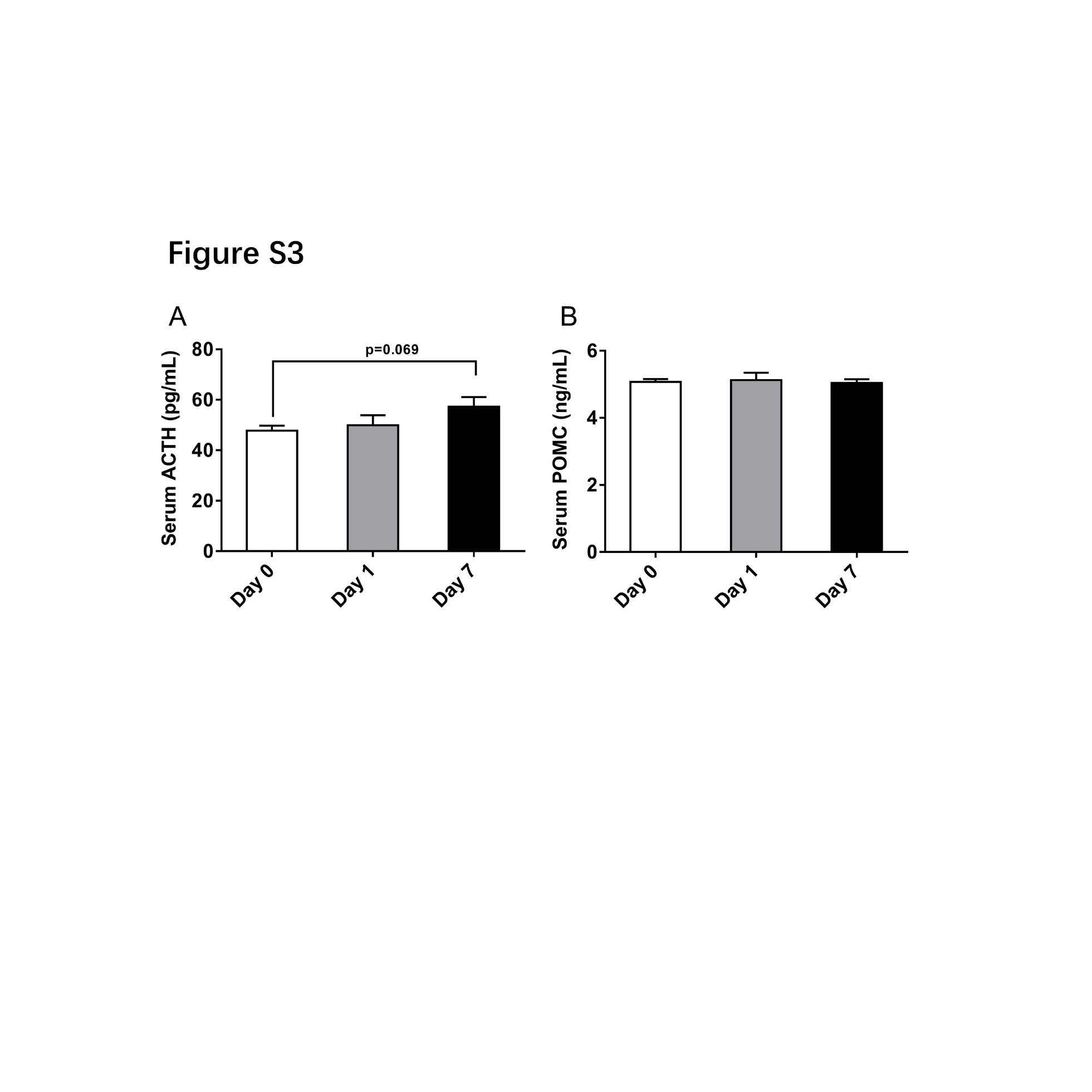

Supplement: Figure S3 — The serum concentrations of ACTH and POMC. The mice of treated groups were placed in 40°C for 2 h each time, and the stimulus, respectively lasted 1 and 7 days. The mice in control group were fed as normal in 25°C. (A) The serum ACTH concentration was detected by Elisa assay. Data are presented as mean ± S.E.M of n = 5 animals per group. Bars that share different letter are significantly different (P < 0.05 by ANOVA). (B) The serum POMC concentration was detected by Elisa assay. Data are presented as mean ± S.E.M of n = 5 animals per group. Bars that share different letter are significantly different (P < 0.05 by ANOVA). [file Image_3.tif]
